# Antenatal Screening for HTLV-1 and -2 Among Pregnant Women in Grenada: Combined Seroprevalence, Trends, and Public Health Implications (2015–2024)

**DOI:** 10.3390/v17111514

**Published:** 2025-11-19

**Authors:** Sherry-Ann N. Joseph, Christine Richards, Yusuf Yakubu, Achut Malur, Tonia Frame

**Affiliations:** 1 Department of Public Health and Preventive Medicine, School of Medicine, St. George’s University, St. George’s P.O. Box 7, Grenada; chriscrich@hotmail.com (C.R.); yyakubu@sgu.edu (Y.Y.); 2 Department of Microbiology, Immunology, and Pharmacology, School of Medicine, St. George’s University, St. George’s P.O. Box 7, Grenada; amalur@sgu.edu

**Keywords:** HTLV, prevalence, adult T-cell leukemia, antenatal, pregnant women, Grenada, Caribbean

## Abstract

The prevalence of Human T-cell Lymphotropic Virus (HTLV) infections in Grenada has not been published since 2013. This study aimed to determine the combined seroprevalence and trends in HTLV-1 and HTLV-2 among pregnant women in Grenada from 2015 to 2024. Data were analyzed to determine the overall combined seroprevalence, observed trends, and public health implications over time. Data obtained from the Ministry of Health, Grenada, were analyzed to determine and compare the annual combined seroprevalence rate and the prevalence by age group and by health district during 2015–2024. Every pregnant woman included in the analysis was tested for HTLV-1 and -2 at the government’s public laboratories in Grenada. The overall rate of infection among persons tested was 1.45%. The highest prevalence of infection was among the 40+ (mature) age group. A significant association was found between HTLV infection and the mature age group (ϰ^2^ = 7.981, *p* = 0.017, OR = 2.6, 95% CI: 1.1559–5.7122). Pregnant women aged 40 years and over are 2.6 times more at risk of infection compared to pregnant adolescents. Trends were also observed by health district, in which the prevalence rate was the highest in St. Patrick (2.18%) and the lowest in St. George (0.95%). Although there were no statistically significant associations observed between HTLV and the COVID-19 pandemic, there is a need for further research to understand the impact of emergencies on HTLV screening and prevalence. Further studies are also needed to identify factors and modes of HTLV transmission. Overall, these findings underscore the importance of targeted surveillance and tailored interventions to address HTLV transmission risks and protect population health in Grenada.

## 1. Introduction

Human T-cell Lymphotropic Virus (HTLV) is an enveloped, single-stranded RNA virus. This member of the Retroviridae family targets T-cells and leads to neurological and hematological diseases [1]. HTLV has at least four serotypes; however, serotypes 1 and 2 have been found to infect humans, with each serotype showing distinct oncogenic properties [2]. HTLV-1 and HTLV-2 have similar genome structures and share about 70% homology, and HTLV-2 is less prevalent than HTLV-1. Additionally, HTLV-1 and HTLV-2 differ in the regulatory and accessory proteins and tissue tropism, with HTLV-1 being primarily detected in CD4^+^ T-lymphocytes and HTLV-2 in CD8^+^ T-lymphocytes [3].

HTLV is primarily acquired through sexual contact with an infected person; however, it can also be transmitted vertically by breastfeeding from an infected mother to her baby, via blood transfusions, or by sharing needles [4]. HTLV is often underdiagnosed, as the associated symptoms, if any, are often absent or appear later in the life of the individual, resulting in an underestimation of the prevalence of the virus [5,6].

Disease manifests in about 5% of people infected with HTLV [7]. It presents as dermatitis, adult T-cell leukemia/lymphoma (ATL), and HTLV-1-associated myelopathy (HAM). ATL is an aggressive type of cancer that results in high mortality [8,9].

Globally, it is estimated that there are 5–10 million people infected with HTLV [10]. HTLV is endemic in countries/regions, such as Japan, South America, and the Caribbean [11]. Among pregnant women in Trinidad and Tobago and French Guiana, the annual prevalence of HTLV was reported as 3–5% [12] and 4% [9], respectively. In the general population of Jamaica, the annual prevalence of HTLV was reported to be 6% [8]. In 2014, Macpherson et al. published an abstract of a retrospective study (1998–2013) demonstrating a 3.9% prevalence of HTLV in the general population of Grenada, from a sample size of 49,738 pregnant women and blood donors [13]. A prevalence of >1% is considered high, as defined by the European Centre for Disease Prevention and Control (ECDC) classification, which means that there is high local transmission of the disease among the population [11].

HTLV is usually diagnosed by blood tests that detect the presence of antibodies to the virus [14]. The confirmatory test that should be used is either PCR/Western blot [15].

Currently, there are no vaccines or specific treatments available for individuals infected with HTLV, and the effectiveness of antiretroviral therapy in managing HTLV is questionable [13,16].

There is currently no regional program in the Caribbean specifically focused on the prevention and eradication of HTLV [17]. Further, there is a lack of prevalence data provided by the ministries of health across Caribbean countries, which hinders effective monitoring. Most of the available data on HTLV comes from blood donor screening and published research articles [17]. However, both the Pan American Health Organization (PAHO) and the World Health Organization (WHO) have identified HTLV prevention as a strategic priority area for 2022–2030 [18,19]. At the regional level, the goal is to reduce mother-to-child transmission by 90–95% by screening all pregnant mothers for HTLV and providing them with information and alternative options to replace breastfeeding [19].

At the global level, WHO has stated that HTLV incidence can be reduced by adopting measures that already exist and has included it in its 2022–2030 strategies [18].

### Antenatal Screening Program

**Figure 1:**
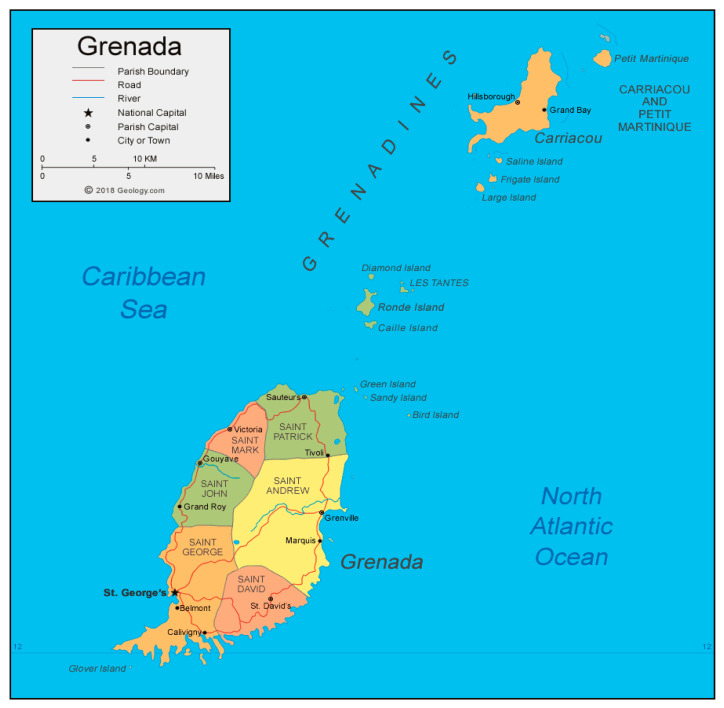
Map of Grenada showing its location and parishes [21].

Grenada is a tri-island nation located to the south of the Caribbean archipelago. It has a population of 124,610 citizens in 2021 [20]. Grenada is divided into seven parishes as shown in Figure 1. The parishes are grouped into six health districts for the administration of health services; the two smaller parishes of St. Mark and St. John are combined into one health district—St. Mark/St. John—and the two smaller islands of Carriacou and Petite Martinique are referred to as the Carriacou health district.

In Grenada, blood samples of pregnant women are collected and tested via routine complete blood count (CBC) and sexually transmitted infection (STI) screening at the first antenatal visit and again at 32 weeks, as part of antenatal care [22]. Screening for HTLV and STIs is offered free to women who attend government antenatal clinics. Midwives in primary healthcare facilities draw blood samples from pregnant women, then send them to the government laboratory for testing. HTLV screening is also offered in private laboratories; however, for this study, the data used are from the government laboratory only.

When a woman’s test is positive, she is counseled by a midwife at her local health center about the implications. These include being unable to breastfeed, getting their partner tested, and alternative feeding options. HTLV-positive mothers are informed about the alternative feeding program offered by the National Infectious Disease Control Unit (NIDCU), which provides supplementary formulas for babies up to age six months. Grenada provides antiretroviral therapy (ART) drugs to people who test positive [22]. However, women who forgo antenatal care are tested at the hospital when they present for delivery or are tested along with their baby post-delivery. Additionally, women who were not screened before delivery are advised not to breastfeed until their HTLV/STI status is determined [22]. District midwives conduct postnatal visits to new mothers to provide support after giving birth. HTLV-positive mothers are counted only once as an incidence and are provided with ART post-delivery [22].

The primary aim of this study is to determine an up-to-date combined seroprevalence of HTLV-1 and -2 infections in pregnant women in Grenada from 2015 to 2024. We hypothesized that there would be no variation in combined seroprevalence based on health district, age, or the presence of the COVID-19 pandemic. 

## 2. Methods

This is a retrospective cross-sectional study that determined the prevalence of HTLV infection in pregnant mothers in Grenada from 2015 to 2024. Prevalence in this study was calculated according to the National Institute of Health definition, where “Prevalence is the proportion of a population who have a specific characteristic in a given time period” [23]. The inclusion criteria were pregnant women whose blood samples were collected and serologically tested for HTLV-1 and -2 during the period 2015–2024. The exclusion criteria were all women who were not pregnant, pregnant women who were not screened for HTLV in both public and private laboratories, and all males.

A convenient sample was used based on the data collected by the Ministry of Health, Epidemiology Division of Grenada. They provided anonymized secondary data for pregnant women whose blood samples were collected and serologically tested for HTLV-1 and -2 during the period 2015–2024. The Government of Grenada data were acquired using two methods: (1) the ICD-10 system for patients seen at the General Hospital level and (2) data from the community-level health facilities and non-government laboratories that are not coded but are acquired at the Central Ministry of Health level [22]. The anonymized datasets contained the number of pregnant women tested, the number of positive tests, the age group, and the parish of residence of the pregnant women at the time of testing. The dataset was not disaggregated into HTLV types but was consolidated as one grouping of HTLV-1 and -2 based on the combined testing protocol established by the Ministry of Health and used by the Government laboratory. The protocol includes the routine testing of complete blood count (CBC) and sexually transmitted infection (STI) screening at the first antenatal visit and again at 32 weeks for samples collected between 2015 and 2024 [22].

Samples are tested using the LIAISON^®^ XL Murex recHTLV-I/II assay from DiaMed DiaSorin. The LIAISON^®^ XL Murex recHTLV-I/II assay is based on the principle of a chemiluminescent assay. It detects HTLV-1 and -2 antibodies using recombinant antigens and peptides. It has a high sensitivity of 100% and a high specificity of 99.94% making it a reliable testing method, and is utilized for detecting HTLV serotypes 1 and 2 worldwide [24].

PCR testing or Western blot is not conducted in the government laboratories; however, once a sample is positive, a second test is performed using the LIAISON^®^ XL Murex recHTLV-I/II assay from DiaMed DiaSorin to confirm the result [22]. This test does not confirm the specific type of HTLV for which the patient tested positive [24].

Ethics approval (20046) was obtained in August 2022 from the Internal Review Board (IRB) at St. George’s University before the data analysis, which began in November 2022 and concluded in July 2025. Informed consent, though valued, was not required for this study. In small island settings, requiring consent for deidentified secondary data may increase the risk of re-identification and compromise confidentiality, especially for individuals with conditions such as HTLV that carry social stigma. Therefore, the Ministry of Health as the custodian of the patient data provided clearance for this study and the deidentified administrative data file without requiring patient consent or patient waiver of consent.

As part of data analysis, the original age categories in the dataset of <15; 15–19; 20–24; 25–29; 30–34; 35–39; 40–44; and ≥45 were recategorized into the following age groups: adolescents <20–; young adults 20–29; older adults 30–39; mature adults ≥ 40+. This recategorization was undertaken to ensure sufficient sample sizes for meaningful calculations, as positivity rates within individual age ranges were either minimal or statistically insignificant. The Ministry of Health data collection is based on the following health districts: St. George, St. David, St. Patrick, St. Andrew, St. John & St. Mark, and Carriacou.

The proportion of HTLV-positive women was calculated as the percentage of women testing positive with the LIAISON^®^ XL Murex recHTLV-I/II Immunoassay out of the total number of pregnant women screened. The proportions of HTLV-positive cases within specific variables were determined by the percentage of HTLV-positive women relative to the total women in each variable. Descriptive statistics were displayed in charts and tables. Chi-square test was used to assess the significant association between demographic variables and the proportion of HTLV-positive women. Negative binomial regression was also used to determine the trend in HTLV prevalence between different age groups from 2015 to 2024. The analysis was performed using R programming software (version 4.3.2). The epitools package was employed in calculating the odds ratios with a significance level of 5% and a 95% confidence interval.

## 3. Results

This study examined blood samples taken from 9967 pregnant women with ages ranging from <15 to >40 years during the period 2015–2024 residing in the state of Grenada. This represents 69.2% of the population of pregnant women in Grenada between 2015 and 2024. Table 1 shows the number of pregnant women screened per age group, per health district, and percentage by year based on the total sample size. Table 2 shows the number of pregnant women screened by age group per health district. 

**Figure 2:**
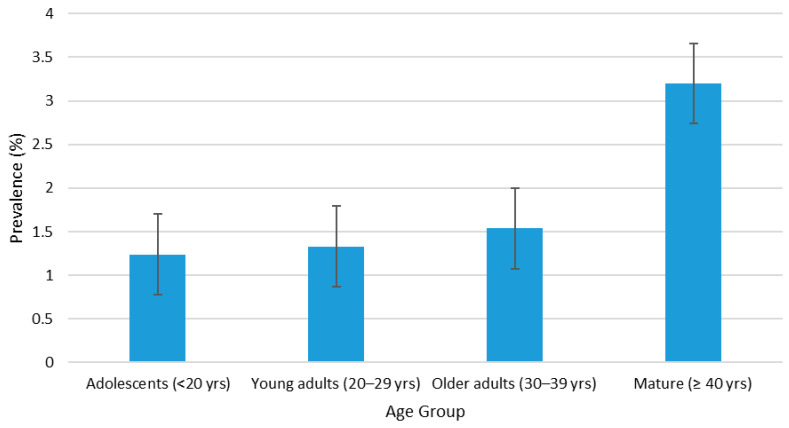
Prevalence of HTLV by age group, 2015–2024.

**Figure 3:**
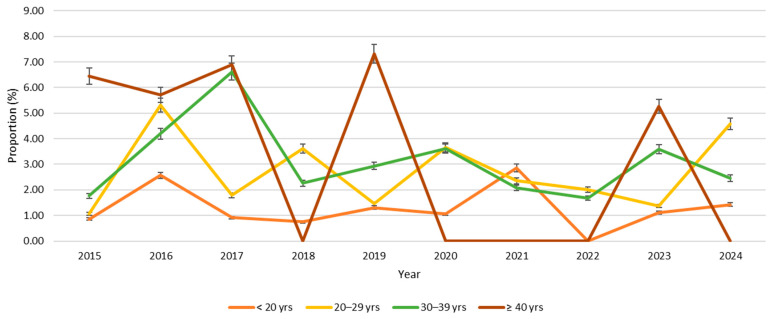
Trends of prevalence of HTLV by age group from 2015–2024.

**Figure 4:**
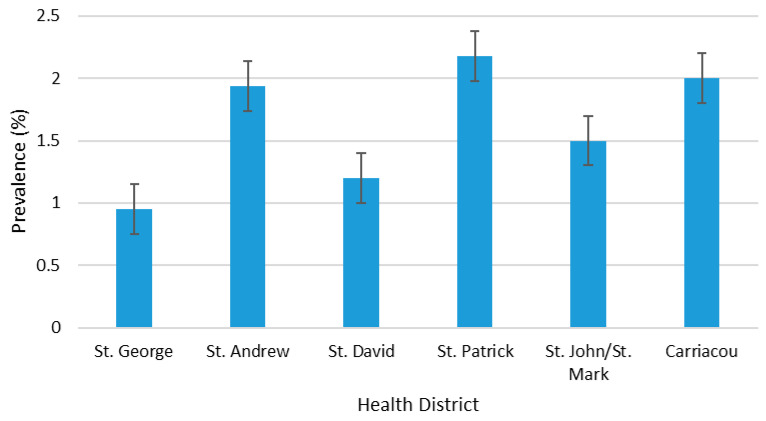
Prevalence of HTLV by health district, 2015–2024.

**Figure 5:**
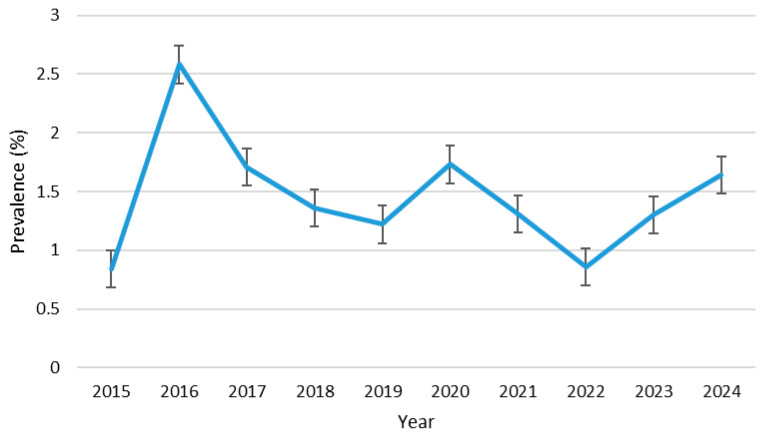
Trend of prevalence of HTLV from 2015–2024.

From this study, the overall combined seroprevalence of HTLV in pregnant women in Grenada during the 10-year period, from 2015 to 2024, was 1.45% (145/9967). Participants who were 40 years and older (mature) had the highest prevalence of 3.20% (11/344), followed by older adults of 1.54% (50/3248), and young adults of 1.33% (70/5250), and adolescents had the lowest prevalence of 1.24% (14/1125) (Figure 2). Similarly, the HTLV prevalence remained relatively low across all age groups, fluctuating between approximately 0% and 8% over the study period. The ≥40 years group exhibited the highest variability, with pronounced peaks in 2016 (6.80%), 2017 (7.0%), and 2019 (7.50%), followed by sharp declines in 2018. However, this age group showed flat fluctuation in seropositivity between 2020 and 2022. In contrast, the <20 years group consistently recorded the lowest prevalence, generally below 3.0%, except for minor increases in 2016, 2021, and 2023, but slightly increased in 2024. The 20–29 years and 30–39 years groups demonstrated intermediate prevalence patterns, ranging between 1.0% and 5.0%, with modest fluctuations and slight upward trends in 2023 and 2024 (Figure 3). HTLV prevalence by health district was highest in St. Patrick (2.18%), followed by Carriacou (2.0%), St. Andrew (1.94%), St. John/St. Mark (1.50%), St. David (1.20%), and St. George with the lowest prevalence (0.95%) (Figure 4). Within the 10-year study period, the highest HTLV prevalence (2.58%) was reported in 2016 and the lowest (0.84%) was reported in 2015. Subsequently, the annual prevalence from 2017 to 2024 was between 1.30% and 1.73% except in 2022 when it dropped to 0.86% (Figure 5).

Statistical analysis showed significant association between HTLV infection and age group (ϰ^2^ = 7.981). Pregnant women aged 40 years and above are 2.6 times more likely to be HTLV-positive compared to pregnant adolescents under 20 years of age (OR = 2.6, 95% CI = 1.16–5.71, *p* = 0.017). The result also showed a significant association between HTLV infection and health district (ϰ^2^ = 16.633). Pregnant women in St. Andrew (OR = 2.07, 95% CI = 1.33–3.24, *p* = 0.001), St. Patrick (OR = 2.3, 95% CI = 1.39–3.84, *p* = 0.001), and Carriacou (OR = 2.2, 95% CI = 0.97–4.34, *p* = 0.049) were twice as likely to test positive compared to those in St. George. Except for the spike observed in 2016 (*p* = 0.003, OR = 3.1, 95% CI = 1.46–7.40, *p* = 0.003), with the proportion of HTLV-positive pregnant women being three times higher than that in 2015, there was no significant trend observed in the 10-year annual HTLV prevalence including the COVID-19 period of 2020–2022 and in the aftermath of Hurricane Beryl in 2024 (Table 3). The annual prevalence was also not significantly different (*p* > 0.05) between the age groups within the 10 years (Table 4).

## 4. Discussion

The main result of this study demonstrates a combined seroprevalence rate of 1.45% of HTLV-1 and -2 in pregnant women in Grenada during 2015–2024. Based on the ECDC classification, 1.45% is categorized as high prevalence [11]. The highest combined seroprevalence was found among pregnant women aged 40+ at 3.2%, and is comparable to other studies [24,25]. The significant association between infection and health district (ϰ^2^ = 16.633) highlights geographical disparities and the need for targeted public health interventions including enhanced screening efforts among adults over age 20 in areas with higher prevalence rates.

The high prevalence of HTLV observed among adults aged 40 and older, compared to adolescents, aligns with existing studies showing age-related increases in seroprevalence, particularly among women [25,26]. This increased prevalence may be attributed to risky sexual behaviors; however, mother-to-child transmission cannot be ruled out, as no infant testing is conducted in Grenada. After the age of 30, most women have had at least one or more sexual partners, increasing the likelihood of acquiring HTLV [24]. Consequently, women over 40 are at a higher risk of transmitting the virus to their babies. Nonetheless, prevalence rates among adolescents are a cause for concern, especially since adolescents represent one of the lowest percentages of pregnant women screened for HTLV. There is a need to explore the factors associated with HTLV transmission among adolescents and to develop interventions targeting this group, given the early age of sexual debut, high teenage pregnancy rates, sexual violence [27,28], and STIs [29] among adolescents in Grenada.

Although HTLV prevalence among pregnant women during the COVID-19 pandemic was not statistically significant, a transient spike in combined seroprevalence was observed in 2020. Since then, screening rates have declined and have not returned to pre-pandemic levels observed in 2018–2019, likely due to reagent stockouts in government laboratories. The cause of the 2020 spike remains unclear. Grenada’s pandemic policies, including movement restrictions, may have influenced access to sexual partners, raising questions about the pandemic’s impact. Caution is advised when interpreting data from 2020 to 2022, as it may not reflect the true combined HTLV seroprevalence. We attribute the decrease in the number of people screened in 2024 to Hurricane Beryl, which severely impacted the northern, eastern, and western regions of the island. The steady increase in seropositivity seen in 2023 and 2024 especially among adolescents (<20 years) and younger adults (20–29 years) may be due to the commencement of targeted STI awareness initiatives during 2023–2024, particularly among adolescents and youth, by the Grenada Planned Parenthood Association (GPPA), which may have contributed to increased detection rates of HTLV even following a disaster period. Similar prevalence trend studies among pregnant women in the Caribbean have not been found in the literature. However, a recent systematic review and meta-analysis of the prevalence of HTLV-1 and -2 infection in pregnant women in Central and South America and the Caribbean, which included studies from 1992 to 2021, found a pooled prevalence of 1.30% (95% CI: 0.96–1.69) using anti-HTLV-1 and -2 antibody screening tests [30]. While this rate is lower than the ten-year combined seroprevalence for Grenada, there is alignment with the broader historical data range suggesting that Grenada’s current prevalence is within the expected variability. Even with regional differences, Grenada’s HTLV prevalence is not an outlier given long-term data trends. Additionally, a study in French Guiana showed a 4% prevalence among pregnant women in 2019 [12].

It is established that HTLV-1 is significantly more prevalent than HTLV-2 across the Caribbean region [31]. For example, a 2022 report by the Pan American Health Organization (PAHO) highlights HTLV-1 prevalence among pregnant women across the Americas, with notably higher rates in several Caribbean and Latin American countries, ranging from 1.9–2.3% in Martinique to 2.2–4.2% in Haiti [32]. Given this context and the lack of confirmatory tests in Grenada required to identify the specific HTLV serotype, it is cautiously assumed that HTLV-1 comprises the larger proportion of HTLV prevalence in Grenada. HTLV-2 is largely confined to specific indigenous populations and intravenous drug users [30], both of which are not commonplace in the Grenada context.

While there is a clear need for trend studies in HTLV-endemic countries across the Americas and for disaggregated HTLV-1 and HTLV-2 data in Grenada, countries like Brazil and Argentina have already implemented screening programs aimed at reducing vertical transmission of the virus [32]. Further afield, Japan saw a reduction in HTLV-1 prevalence by the early 1990s and prevents approximately 80% of potential mother-to-child transmissions [33]. Japan saw a decrease by testing all pregnant women for HTLV, reducing the number of mothers who breastfeed and shortening the breastfeeding period [33]. These efforts highlight the importance of proactive public health strategies.

The significant association between HTLV infection among pregnant women and parish of residence is important. On mainland Grenada, St. Andrew and St. David are located in the east, St. Patrick in the north, and St. John/St. Mark in the west. Carriacou, a smaller separate island, lies north of the mainland. All of these parishes are considered rural. In contrast, St. George in the south is the only urban parish. This finding suggests that there may be a geographic clustering of HTLV in rural areas. For example, St. Patrick’s parish had the highest prevalence rate of 2.18% compared with the other parishes and the national prevalence rate. Nationally, St. Patrick’s parish is reported to have one of the highest poverty rates, and in 2008, 56.7% of residents were classified as “poor” based on living conditions and household budgets [27]. Other studies have shown that poverty and lower education levels are associated with inconsistent condom use and unprotected sex, which increases the chances of contracting sexually transmitted infections, including HTLV [33,34], and continuation of breastfeeding [35]. The variations observed in geographic and age group prevalence highlight the complexity of HTLV prevalence in Grenada, suggesting that a one-size approach to HTLV prevention would most likely be inadequate [33,34]. With national and health district-level combined HTLV seroprevalence exceeding 1%—except in St. George—targeted prevention strategies are needed both nationally and locally, focusing on adults aged 35+ while also addressing all age groups given the widespread prevalence.

While sociodemographic characteristics were not examined in this study, one study in Brazil amongst pregnant women showed that a greater population of HTLV-1-positive women were underemployed [36]. It would be beneficial if these type of data became available in Grenada in the future, given that poverty is higher in rural areas compared to urban areas in Grenada [35]. This would mean that education and screening initiatives can be targeted to particular groups of women.

Grenada can emulate Japan’s success by implementing comprehensive protocols, including the routine screening of pregnant women and confirmatory testing using methods like Western blot and PCR. Early detection facilitates timely interventions, such as advising HTLV-positive mothers to refrain from breastfeeding. Additionally, adopting Japan’s strategy of targeting “hot spots” can be beneficial for Grenada, particularly in areas like St. Patrick, Carriacou, and among the 40+ population, where prevalence is the highest. Further measures include discouraging breastfeeding among HTLV-positive mothers and mandating HTLV testing for blood donors. Public awareness initiatives and training healthcare professionals to communicate prevention strategies effectively have contributed to a sustained decrease in HTLV transmission rates, demonstrating the effectiveness of Japan’s integrated public health management approach [37]

The findings from the current study can inform the development of more targeted research interventions aimed at addressing problematic areas and refining testing protocols. This information can enhance surveillance methods employed by regional and international agencies such as Caribbean Public Health Agency (CARPHA), PAHO, and WHO. The findings support the hypothesis that HTLV seroprevalence among pregnant women in Grenada varies by health district and age, rejecting the null hypothesis. However, no significant variation was observed during the COVID-19 pandemic, which supports the null hypothesis. Additionally, it highlights the necessity of employing consistent and reliable testing methods to ensure accurate prevalence data, which is essential for developing effective public health strategies.

### 4.1. Limitations of This Study

A major limitation of this study was the use of administrative data with limited parameters for statistical analysis. The aggregated data compiled by the Ministry of Health do not capture the exact age of the women and other relevant information, such as place of residence, socioeconomic status, marital status, spouse’s HTLV status, employment history, etc. This gap has limited the hypothesis testing to determine more potential risk factors associated with the infection in pregnant women screened. Also, during the study period, a total of 14,400 pregnancies were reported. However, only 9967 of the pregnant women were tested for HTLV, meaning results were unavailable for almost one-third (30.8%) of pregnant women who were tested This gap may have implications for accurately determining the true combined HTLV seroprevalence in Grenada.

The use of a convenient sample means that the sample was based on availability and not a representative sample of the larger population, thus creating selection bias, limiting the ability to generalize the results to other populations. For example, stockouts of reagents would have hindered testing of pregnant women in government health facilities; thus, the true context of testing in the government laboratories could not be presented.

This study looks at screening tests and not confirmatory tests. This means that the data reflect the prevalence of screening test results only and limits generalizability of the findings to countries like Trinidad, Jamaica, Brazil, and Japan, where confirmatory tests are utilized. PAHO currently recommends PCR testing to be employed in Caribbean countries. Countries were advised to pool resources and conduct training where applicable to perform confirmatory testing [15]. Potential misdiagnosis may lead to unwarranted spending on ARV drugs and alternative feeding formulas. Additionally, if mothers are HTLV-positive, they will need to confirm this to understand the future implications not only for future pregnancies but for the mother’s own health.

Additionally, this study categorized all cases under age 15 as within the adolescent group, which could have affected the prevalence among this population. However, few pregnancies under age 16 are recorded in Grenada. Finally, the unavailability of data on modes of transmission restricted the analysis of trends.

### 4.2. Recommendations

1. Confirmatory Testing Improvements
  - Implement PCR or Western blot methods in the government laboratories to reduce false positives and accurately assess HTLV prevalence.
  - Report positive cases by HTLV serotype to better understand prevalence and associated diseases.
2. Antenatal Clinic Initiatives
  - Encourage antenatal visits for all pregnant women to ensure compliance with HTLV testing.
  - Educate healthcare providers and seropositive mothers on the importance of discontinuing breastfeeding.
  - Continue the alternative breastfeeding program, providing formula to HTLV-positive mothers, like the successful HIV program.
3. Education and Awareness
  - Integrate HTLV education into Grenada’s Baby Friendly Hospital Initiative (BFHI) to sensitize mothers about the virus and transmission routes.
  - Improve HTLV awareness as an STI through government, NGO, and private sector initiatives, including school education programs.
4. Population Testing and Registry
  - Create a national program for HTLV as that created for HIV, with the following:
    - Collaborate with researchers and donor agencies to determine HTLV prevalence in the general population.
    - Include HTLV testing in routine STI panels for “at-risk” populations and those requesting STI screening.
    - Establish a population-based STI registry inclusive of HTLV to track trends and individuals developing related diseases.
5. Targeted Interventions
  - Develop HTLV and STI prevention programs promoting safe sex, especially for individuals with multiple partners.
  - Offer HTLV testing to broader populations, including men, as part of routine STI screening for men with multiple partners.
  - Offer HTLV testing in “hot spot” areas where prevalence is the highest.
  - Test babies born to HTLV-positive mothers.
6. Focus on the Highest Prevalence Areas and Sub-populations
  - Target interventions in St. Patrick’s parish (2.18% prevalence) and mature adults in St. David’s parish (11.99% prevalence).
  - Address adolescent prevalence in St. John/St. Mark and Carriacou (4.29% and 2.78%, respectively).
7. Further Research
  - Conduct studies to assess HTLV prevalence and transmission risk factors among the general population, pregnant women, and men, as well as during health and other emergencies, and utilize confirmatory testing that can determine HTLV serotypes. Include datasets from private laboratories to ensure broader and more representative population coverage. Additionally, integrating data from both private and public laboratories will enable the detection of cross-infection. This approach will ensure a more comprehensive understanding of HTLV prevalence and facilitate more effective deployment of interventions.
  - Replicate studies in other Caribbean countries and among Caribbean populations to assess prevalence and intervention needs.

## 5. Conclusions

This is the first study undertaken in Grenada and the Eastern Caribbean that examines the combined HTLV-1 and HTLV-2 seroprevalence and trends among pregnant women, along with its associated public health implications. The results show a high combined seroprevalence rate of 1.45% of HTLV-1 and -2 in pregnant women in Grenada during 2015–2024, with higher prevalence observed among older age groups and across specified geographic regions. These disparities and fluctuations observed in screening rates and seropositivity highlight the need for targeted and sustained public health interventions, informed by successful models such as those implemented in Japan and aligned with PAHO/WHO recommendations. Implementation of these strategies may help to reduce the transmission of HTLV among pregnant women, mitigate vertical transmission, and ultimately decrease HTLV-related morbidity and mortality.

The potential negative impact of emergency and crisis situations on HTLV screening and prevalence due to disrupted service delivery remains unclear, but warrants further investigation to understand the long-term implications, particularly given Grenada and the Caribbean’s vulnerability to health (e.g., COVID-19) and climate shocks (e.g., Hurricane Beryl). Strengthening health system resilience and integrating HTLV screening into broader emergency preparedness frameworks might be critical to safeguarding public health in the Caribbean region.

To improve diagnostic accuracy and better understand HTLV epidemiology, confirmatory testing, at minimum, using Western blot but ideally PCR as the gold standard should be prioritized to exclude false positives and differentiate between HTLV serotypes, which will allow for comparisons with other contexts. Further research should explore population-specific risk factors through qualitative research and incorporate data from private laboratories to ensure comprehensive surveillance and response.

## Figures and Tables

**Table 1:** Demographic profile of women screened for HTLV in Grenada from 2015–2024.

Variable	Level	No. of Pregnant Women	Percentage (%)
Age group	Adolescents (<15–19 years)	1125	11.29
	Young adults (20–29 years)	5250	52.67
	Older adults (30–39 years)	3248	32.59
	Mature adults (≥40 years)	344	3.45
Health District	St. George	3796	38.09
	St. Andrew	2265	22.72
	St. David	1414	14.19
	St. Patrick	1241	12.45
	St. John/St. Mark	802	8.05
	Carriacou	449	4.50
Year	2015	958	9.61
	2016	1047	10.50
	2017	993	9.96
	2018	1251	12.55
	2019	1313	13.17
	2020	1039	10.42
	2021	841	8.44
	2022	934	9.37
	2023	920	9.23
	2024	671	6.73

**Table 2:** Distribution of women screened by age group per health district.

Health District	Adolescents (<20 Years)	Young Adults (20–29 Years)	Older Adults (30–39 Years)	Mature Adults (≥40 Years)
St. George	389	1994	1281	132
St. David	172	707	495	40
St. Andrew	272	1257	655	81
St. Patrick	156	672	366	47
St. John/St. Mark	90	401	281	30
Carriacou	46	219	170	14

**Table 3:** Univariable analysis for the association between HTLV infection and selected variables.

Variable	Level	No. Positive (%)	ϰ^2^ Value	*p*-Value	Odds Ratio	95% CI	
Age group	Adolescents (<20 years)	1.24(14/1125)	7.981	Ref	1.0	NA	NA
	Young adults (20–29 years)	1.33(70/5250)		0.815	1.0714	0.6014	1.9087
	Older adults (30–39 years)	1.54(50/3248		0.484	1.2370	0.6813	2.2461
	Mature adults (≥40 years)	3.20(11/344)		0.017	2.5695	1.1559	5.7122
Health DistrictPari	St. George	0.95(36/3796)	16.633	Ref	1.0	NA	NA
	St. David	1.20(17/1414)		0.417	1.2773	0.6959	2.2515
	St. Andrew	1.94(44/2265)		0.001	2.0672	1.3267	3.2419
	St. Patrick	2.18(27/1241)		0.001	2.3259	1.3915	3.8417
	St. John/St. Mark	1.5(12/802)		0.179	1.6005	0.7914	3.0100
	Carriacou	2.00(9/449)		0.049	2.1643	0.9666	4.3428
Year	2015	0.84(8/958)	16.164	Ref	1.0	NA	NA
	2016	2.58(27/1047)		0.003	3.0981	1.4586	7.3978
	2017	1.71(17/993)		0.085	2.0461	0.8987	5.0911
	2018	1.36(17/1251)		0.249	1.6185	0.7113	4.0255
	2019	1.22(16/1313)		0.377	1.4502	0.6301	3.6306
	2020	1.73(18/1039)		0.077	2.0698	0.9186	5.1193
	2021	1.31(11/841)		0.328	1.5650	0.6233	4.1161
	2022	0.86(8/934)		0.959	1.0259	0.3700	2.8444
	2023	1.30(12/920)		0.322	1.5587	0.6351	4.0478
	2024	1.64(11/671)		0.137	1.9677	0.7831	5.1784

Ref: reference category; NA: not applicable; CI: confidence interval.

**Table 4:** Negative binomial regression for the trend and association between HTLV infection and age group from 2015–2024.

Variable	Level	*p*-Value	Odds Ratio	95% CI	
Age group	Adolescents (<20 years)	Ref	1.0	NA	NA
	Younger adults (20–29 years)	0.760	1.18	0.390	3.460
	Older adults (30–39 years)	0.628	1.31	0.440	3.870
	Mature adults (≥40 years)	0.150	2.46	0.720	8.340
Year	2015	Ref	1.0	NA	NA
	2016	0.316	2.31	0.450	11.800
	2017	0.510	1.76	0.340	9.360
	2018	0.965	0.96	0.190	4.910
	2019	0.687	1.41	0.280	7.090
	2020	0.775	1.28	0.250	6.570
	2021	0.826	1.21	0.230	6.280
	2022	0.591	0.61	0.120	3.090
	2023	0.765	1.30	0.260	6.570
	2024	0.800	1.25	0.240	6.440

Ref: reference category; NA: not applicable.

## Data Availability

Data can be found in the Department of Epidemiology, the Ministry of Health, Grenada.

## Abbreviations

The following abbreviations are used in this manuscript:

HTLV	Human T-Lymphotropic Virus
HIV	Human Immunodeficiency Virus
STI	Sexually Transmitted Disease
ATL	Adult T-cell Leukemia/Lymphoma
PAHO	Pan American Health Organization
COVID-19	Coronavirus Disease of 2019
ICD-10	International Classification of Diseases
WHO	World Health Organization
NIDCU	National Infectious Disease Control Unit
BFHI	Baby Friendly Hospital Initiative
CARPHA	Caribbean Public Health Agency
